# The impact of role conflict on turnover intention among faculty members: A moderated mediation model of emotional exhaustion and workplace relational conflict

**DOI:** 10.3389/fpsyg.2022.1087947

**Published:** 2022-12-21

**Authors:** Ahmed M. Asfahani

**Affiliations:** Department of Human Resources Management, University of Business and Technology, Jeddah, Saudi Arabia

**Keywords:** role conflict, turnover intention, relational conflict, emotional exhaustion, moderated mediation model

## Abstract

This study examines the impact of emotional exhaustion on faculty role conflict as a source of stress that leads to turnover intention, which is considered to be an undesirable organizational behavior. Drawing on conservation of resources and job-demand theories, the study investigates the moderating effect of workplace relational conflict on the relationships between role conflict among faculty members and both emotional exhaustion and turnover intention. Cross-sectional survey data were collected from 321 faculty members employed in 58 Saudi universities and institutions of higher education. Structural equation modeling revealed that emotional exhaustion mediates the significant positive relationship between faculty members’ role conflict and their turnover intention and that these mediating effects are enhanced by the moderating effect of workplace relational conflict on the significant positive relationship between emotional exhaustion and turnover intention. Unexpectedly, the moderating effect of workplace relational conflict on the relationship between role conflict and emotional exhaustion was not supported by the results. The study concludes by highlighting several theoretical and practical implications and providing suggestions for future research.

## Introduction

Due to the current dynamic and competitive nature of society, the higher education sector worldwide faces several extraordinary managerial, financial, and operational challenges (Neuwirth et al., 2021). These changes and challenges have contributed to the increasing demands for the creation of a better academic environment that can meet the needs and satisfy the interests of all academic stakeholders, including students, administrators, and faculty members, to maintain and improve institutional productivity (Sabagh et al., 2018). In addition, human resources management in the context of educational institutions plays an important role in contributing to the achievement of the desired objectives of those institutions and their ability to obtain competitive advantage (Tyson, 1995). It is the responsibility of human resources management to provide a better work environment for faculty members, including by maintaining and ensuring their well-being, managing their role and workplace relational conflicts, creating balanced job designs and job descriptions for them, and retaining the best faculty members.

In recent years, the importance of role conflict among faculty members has attracted the interest of scholars, who have investigated this issue by reference to a variety of different variables, including professional and academic development (de Wildermuth et al., 2022; Hakkola and Dyer, 2022), organizational support (Richards et al., 2021), satisfaction (Zhang et al., 2018; Tahir et al., 2022), absenteeism (Olivares-Faúndez et al., 2014), and burnout (Xu, 2017). Typically, faculty members have three main key responsibilities or roles, namely, teaching, research and community engagement (Fernandez, 2020). The relationships among these different yet interdependent roles are indivisible and reciprocal (Shawa, 2019). That is, faculty members should pay equal attention to each role to satisfy their job demands. However, conflict among those roles is inevitable due the lack of the time and energy that are necessary to handle faculty members’ various duties and responsibilities, which can lead to negative emotional and physical outcomes (Moore, 1963; Ladd, 1979; Eble, 1994). Consequently, the more that faculty members focus on one role, the less attention they pay to the other two roles. Specifically, role conflict can force faculty members to balance their time and available resources, in which context they may become distracted by the compensation differences among faculty members. One of the most critical consequences of role conflict is emotional exhaustion (Edú-valsania et al., 2022).

Emotional exhaustion refers to a feeling that is characterized by chronic fatigue and low energy and is considered to be one of the main dimensions of burnout (Bui et al., 2022). Several previous studies (Jin et al., 2016; Chun et al., 2022; Jeong and Lee, 2022; Yang et al., 2022) have explained the relationship between role conflict and emotional exhaustion from the perspective of conservation of resources (COR) theory (Hobfoll, 1989). Due to the negative emotions resulting from role conflict, faculty members find themselves unable to control and balance the demands of each role, resulting in energy depletion. Emotional exhaustion not only affects faculty members but also has negative consequences for the organization. One of the most crucial organizational concerns is the possibility of losing talent and potential turnover intention among talented individuals due to their high levels of emotional exhaustion (Wen et al., 2020). The negative consequences of turnover intention for organizations include workforce instability (Strouse and Reed, 2021), productivity reduction (Newsham et al., 2022), counterproductive behavior (Hattab et al., 2022), and cost increases (Söylemez, 2021). However, little attention has been given to the task of understanding the ways in which the impacts of role conflict among faculty members could affect their turnover intention in general or among Saudi faculty members in particular.

To address this research gap, this study took faculty members in Saudi universities and institutions of higher education as its research subjects to investigate the relationships among a variety of relevant variables, including role conflict, emotional exhaustion, turnover intention, and workplace relational conflict. In addition, the study explores the moderating and mediating effects on the relationships among those variables. In particular, the current study investigates the ways in which workplace relational conflict alters the relationship between role conflict and emotional exhaustion as well the relationship between role conflict and turnover intention. Previous studies have highlighted the fact that workplace relational conflict negatively affects faculty members’ ability to cope with the role conflict they experiences (Zainun et al., 2018; Krajcsák, 2022; Kundi et al., 2022), increases their turnover intention (Vui-Yee and Yen-Hwa, 2020; Akhlaghimofrad and Farmanesh, 2021; Counts et al., 2021), and increases their emotional exhaustion (Madigan and Kim, 2021; Ghasemi et al., 2022; Wray and Kinman, 2022). To the best of the researcher’s knowledge, no study has investigated the conceptual model proposed by the current study, which aims to address this ongoing gap in the literature.

This study was divided into several sections. The conceptual framework and study context are provided by the theoretical framework and literature review. Moving from the conceptual to the concrete, the methodology section describes the procedures and research instruments utilized to achieve the findings. The paper finishes with a discussion of numerous practical consequences and suggestions for further research.

## Literature review and hypothesis development

### Employee turnover and turnover intention

Employee turnover can be described as the ratio between the number of employees who leave an organization and the average number of employees in the organization over the same timeframe (Price, 1977). Employee turnover ras be divided into two main categories based on the initiator of the process: voluntary and involuntary. Voluntary turnover refers to situations in which employees decide to end their relationship with their organizations (Liborius and Kiewitz, 2022), whereas involuntary turnover occurs when employers decide to terminate or refrain from renewing their employment relationship with their employees (Burrows et al., 2022). Irrespective of these categories, employee turnover has always been a matter of concern for organizations due to its significant effects on organizations in terms of organizational performance (Wynen et al., 2018), financial cost (Friedman and Neutze, 2020), knowledge (Lakshman et al., 2022), and loss of skills (Dhar et al., 2022).

In this respect, turnover intention indicates employees’ awareness of the possibility of and willingness to leave the organization in the near future and is usually viewed as a predictor of actual employee turnover (Mowday et al., 1982; Lazzari et al., 2022). By understanding the antecedents of turnover intentions, organizations could prevent the actual turnover of their valued employees by modifying organizational policies and procedures with the aim of retaining those employees (Jabeen et al., 2020; Park and Min, 2020; Li and Yao, 2022). To measure turnover intention effectively, Mobley et al. (1978) identified three relevant dimensions, namely, attitudinal (thinking of quitting), decisional (intending to leave), and behavioral (searching for a new job).

### Role conflict and its relation to turnover intention

Role conflict refers to a situation in which role expectations are ambiguous or mutually exclusive (Kahn et al., 1964; Hardy, 1988). Due to the rapid development of the academic environment and academic institutions, the role of faculty members has also become more complex (Davis, 1995). Typically, faculty members’ roles encompass teaching, research, and service to the community (Fernandez, 2020). In addition to those main roles, other faculty members might face additional administrative and managerial demands (Sobrero and Jayaratne, 2014; Gottardello and Karabag, 2020; Swann et al., 2021). Several studies have shown that the multiplicity of faculty members roles leads to stress, burnout, and role conflict (Xu, 2017; Nassar et al., 2019; Li et al., 2020). For example, role conflict occurs when a faculty member faces an ambiguous choice between focusing on conducting high-quality academic research or improving his or her teaching techniques.

Previous studies on role conflict have predominantly examined its impact on employees’ turnover intentions and found that role conflict acts as a dissatisfier and stressor with regard to employees that can motivate them to leave their organizations (Miller and Anderson, 2012; Ainer et al., 2018; Cho et al., 2019; Gilani and Rabbani, 2020; Rangrez et al., 2022; Salama et al., 2022). This relationship can be explained by resource theories such as COR theory (Hobfoll, 1989). According to COR theory, stress arises when individuals are endangered by resource loss, and to prevent further loss of resources, exhausted individuals develop withdrawal coping mechanisms. Therefore, due to the stress and exhaustion resulting from role conflict, faculty members might feel relucent to invest additional resources into their jobs and might begin considering and seeking alternative careers or better job opportunities (Jin et al., 2016; Chun et al., 2022; Jeong and Lee, 2022; Yang et al., 2022). Based on this argument, the researcher proposes the following hypothesis:

*H1*: There is a significant direct positive association between role conflict and turnover intention among faculty members in Saudi Arabia.

### The mediating role of emotional exhaustion

Defined as a state in which the individual feels fatigued, frustrated, and worn out due to the continuous depletion of emotional resources from overextended work (Boles et al., 1997; Thompson et al., 2020; Dinc et al., 2022), emotional exhaustion is now considered to be a problem worldwide and is associated with employees’ work-related burnout (Ayachit and Chitta, 2021; Karuna et al., 2022). In this context, faculty members feel emotionally exhausted, which affects their ability to control and reduce their ability to cope with the job demands they face (Cordes and Dougherty, 1993). Since emotional exhaustion is considered to be a core dimension of burnout, when a faculty member experiences burnout, they also experience emotional exhaustion (Abdelmounaim et al., 2022).

*H2*: There is a significant direct positive association between emotional exhaustion and turnover intentions among faculty members in Saudi Arabia.

In terms of the frameworks of COR theory (Hobfoll, 1989) and job demand-resources (JD-R) theory (Demerouti et al., 2001), negative situations deplete valued resources, and employees attempt to reduce threats and prevent the potential loss of those resources. Such depletion prevents faculty members from coping with tense and negative events such role conflict, thus leading to emotional exhaustion due to their continuous attempts at resource protection (Hsieh et al., 2021; Bakker et al., 2022). A substantial amount of research has investigated the mediating role of emotional exhaustion in the relationships between different stressor variables, such as abusive supervision (Ali et al., 2022), psychological empowerment (Ding, 2021), job crafting (Shin et al., 2020), and role stress (Wen et al., 2020), and employees’ turnover intention. Based on the preceding discussion, the researcher proposes the following hypothesis:

*H3*: The relationship between role conflict and turnover intention among faculty members in Saudi Arabia is mediated by emotional exhaustion.

### The moderating role of workplace relational conflict

Relational conflict is a relatively new term that refers to the awareness of individual and emotional incompatibilities within teams or groups; such conflict occurs when individuals exhibit aggression and resentment toward each other, which can lead to hostile and anxious interactions among them (Jehn, 1994, 1995; Jehn and Mannix, 2001). Most scholars have claimed that relational conflict leads to negative consequences for organizational performance and teams’ work-related outcomes (Spector and Jex, 1998; De Dreu and Van Vianen, 2001; Krueger et al., 2022). Previous research has indicated a potential association between workplace interpersonal conflict and a variety of negative emotional outcomes, including counterproductive work behavior (Kundi and Badar, 2021), psychological strain (Somaraju et al., 2022), and emotional exhaustion (Anjum et al., 2020).

As indicated previously, faculty members can experience negative emotions on the job due to their different stressful and demanding job roles, which requires them to employ coping strategies to prevent or minimize such exhaustion (Frone, 2000; Wright and Larson, 2022), thus consuming their resources (Hsieh et al., 2021; Bakker et al., 2022). Since those resources are limited, the regular demands of workplace relational conflict may compete with the regular demands of role conflict. Such competition is expected to decrease the resources available to faculty members, which are required to control the negative emotions associated with their role conflict; and this situation makes faculty members more likely to experienced increased emotional exhaustion. Furthermore, the resources available to faculty members are expected to be less threatened if those faculty members experience less workplace relational conflict, which can increase their ability to cope with the emotional exhaustion caused by their role conflict, as their resource depletion is less likely in this context. Hence, the following hypotheses are proposed:

*H4*: Workplace relational conflict moderates the relationship between role conflict and emotional exhaustion among faculty members such that this relationship is weaker when interpersonal conflict is low.

*H5*: Workplace relational conflict moderates the relationship between role conflict and turnover intention among faculty members such that this relationship is stronger when interpersonal conflict is high.

**Figure 1:**
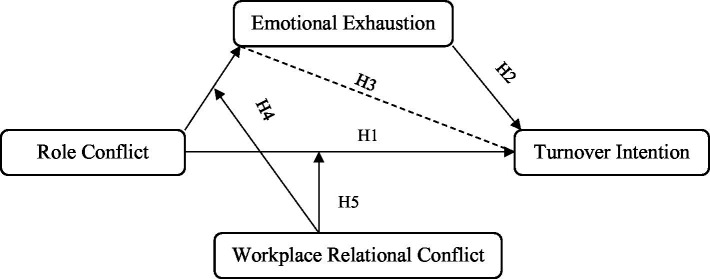
The theoretical model.

Thus, this study presents its theoretical model based on the proposed hypothesis in Figure 1.

## Materials and methods

### Procedure and participants

The researcher collected data from faculty members working in Saudi universities, colleges, and institutions of higher education. An online survey was created, and an invitation to participate in a voluntary 15-min online survey was sent to 1,000 faculty members; the researcher promised to keep their data confidential. With the aim of improving the response rate, the researcher sent participants reminder emails containing a promise that a summary of the findings would be shared with them. In total, 321 valid responses were received, for a response rate of 32.1%; the participants included 59.8% males, 48.6% of participants held the rank of assistant professor, 80.4% of participants were married, and 47% of participants were between 35 and 44 years old. Table 1 provides more details regarding the representativeness of the sample.

**Table 1:** Sociodemographic characteristics of the participants.

Sample characteristics	*N*	%
Gender		
Male	192	59.8
Female	129	40.2
Age		
25–34	62	19.3
35–44	151	47.0
45–54	80	24.9
55–64	25	07.8
65+	03	00.9
Academic rank		
Professor	18	05.6
Associate professor	42	13.1
Assistant professor	156	48.6
Lecturer	105	32.7
Marital status		
Married	258	80.4
Divorced	12	03.7
Single	51	15.9

*N* = 321.

### Measures

Measures of the study variables were adopted from established instruments discussed in the previous literature. Double-blind back translation from the English language to the Arabic language was employed to ensure conceptual equivalence (Brislin, 1980). Four items measuring role conflict were drawn from the teacher/coach role conflict scale developed by Ryan (2008), which included response categories ranging from 1 (strongly disagree) to 7 (strongly agree). In addition, 4 items were drawn from Bothma and Roodt (2013) to measure faculty members’ turnover intentions; responses to these items were scored on a 7-point Likert scale ranging from 1 (strongly disagree) to 7 (strongly agree). To measure faculty members’ emotional execution, the researcher chose 4 relevant items developed by Tate et al. (1997). In addition, a subscale developed by Doucet et al. (2009) was used to measure faculty members’ workplace relational conflict on a 7-point Likert scale ranging from 1 (strongly disagree) to 7 (strongly agree). As recommended by several scholars (Breaugh, 2008; Carlson and Wu, 2011; Becker et al., 2016), the researcher also used gender as a control variable due to its theoretical ties with role conflict (Boles et al., 2003). The survey’s overall Cronbach’s α was 0.919, indicating a very good level of reliability (Hulin et al., 2001).

### Reliability and validity

Exploratory factor analysis (EFA) was conducted to check dimensionality and to assess the interrelationships among the constructs (Pituch and Stevens, 2016). As shown in Table 2, the results indicated that all items had higher loadings on their corresponding construct and lower loadings on unrelated constructs. In addition, Cronbach’s α values ranged from 0.849 to 0.923, indicating high internal consistency and a very good level of reliability for the constructs (Hulin et al., 2001; Fornell and Larcker, 2018).

**Table 2:** Construct reliability and validity analysis.

Construct and items	Factor loadings	S. E	*T* value	Reliability and validity
Role conflict (Ryan, 2008)				
RC 1	0.733	0.112	37.264	Cronbach’s α = 0.822 CR = 0.839 AVE = 0.568
RC 2	0.798	0.111	38.359	
RC 3	0.827	0.110	28.398	
RC 4	0.644	0.117	38.027	
Emotional exhaustion (Tate et al., 1997)				
EE 1	0.727	0.111	24.843	Cronbach’s α = 0.861 CR = 0.880 AVE = 0.648
EE 2	0.833	0.112	30.821	
EE 3	0.842	0.115	25.689	
EE 4	0.812	0.112	17.847	
Turnover intention (Bothma and Roodt, 2013)				
TI 1	0.803	0.126	34.791	Cronbach’s α = 0.900 CR = 0.881 AVE = 0.650
TI 2	0.875	0.129	32.033	
TI 3	0.734	0.130	31.481	
TI 4	0.807	0.122	31.465	
Workplace relational conflict (Doucet et al., 2009)				
WRC 1	0.899	0.093	53.489	Cronbach’s α = 0.894 CR = 0.902 AVE = 0.821
WRC 2	0.914	0.091	55.922	

S. E = Standard error of the mean; CR = Composite reliability; AVE = Average variance extracted.

Confirmatory factor analyzes (CFAs) were conducted to assess the discriminant validity and convergent validity of the study constructs (Becker et al., 2016). The CFA results for the research model (χ2/df = 2.674, RMSEA = 0.072, GFI = 0.927, IFI = 0.957, CFI = 0.957, TLI = 0.944) indicated an acceptable level of model fit (Hooper et al., 2008; Hu and Bentler, 2009). Table 2 presents the summary statistics for the values of average variance extracted (AVE), the standardized factor loadings, composite reliability (CR), and the factor loadings. With factor loadings ranging from 0.602 to 0.914, CR ranging from 0.839 to 0.902, and AVE values ranging from 0.568 to 0.821, the researcher could argue that the constructs used in this study satisfied the criteria for convergent validity (O’Leary-Kelly and Vokurka, 1998). In addition, the discriminant validity was confirmed by comparing the square root of the AVE value for each construct with the correlations among the paired constructs (Fornell and Larcker, 2018), as shown in Table 3.

**Table 3:** Descriptive statistics, correlations, and square roots of AVE values.

Construct	Mean	S.D.	RC	EE	TI	WRC
RC	4.01	1.63	**0.754**			
EE	11.14	6.75	0.575**	**0.805**		
TI	4.11	1.99	0.565**	0.622**	**0.806**	
WRC	5.04	1.57	0.458**	0.350**	0.540**	**0.906**

Square root of AVE is listed on the diagonal in bold. **Correlation is significant at the 0.01 level (2-tailed).

### Data analysis

This study used SPSS 28.0 software and its extension PROCESS, which were developed by Hayes (2013), to analyze the data. PROCESS model 4 was used to test both the first and second hypotheses regarding the relationships between role conflict and turnover intention as well as the relationships between emotional exhaustion and turnover intention. In addition, the same model was used to test the third hypothesis concerning the mediating effect of workplace interpersonal conflict. Moreover, PROCESS model 8 was used to test the fourth and fifth hypotheses pertaining to the moderating effect of workplace relational conflict on the relationship between role conflict and emotional exhaustion as well its moderating effect on the relationship between role conflict and emotional exhaustion.

## Results

As shown in Table 4 and as expected, there is a significant positive relationship between role conflict and turnover intention among faculty members (*β* = 0.377, *p* < 0.001), which supports the first hypothesis proposed by this study. Faculty members who experience role conflict are more likely to express turnover intentions. In addition, the results indicated a positive correlation between emotional exhaustion and turnover among faculty members (*β* = 0.130, *p* < 0.001), which supports the second hypothesis proposed by the current study. That is, the higher the level of emotional exhaustion among faculty members is, the stronger turnover intentions they have. Furthermore, the results supported the third hypothesis proposed by this study, as the relationship between role conflict and turnover intention found to be mediated by emotional exhaustion (indirect effect: *β* = 0.298, *p* < 0.001; direct effect: *β* = 0.377, *p* < 0.001; and total effect: *β* = 0.675, *p* < 0.001). Faculty members who are emotionally exhausted exhibit stronger turnover intentions than those who are not.

**Table 4:** Testing the mediating effect of role conflict on turnover intention.

Predictor	Model 1 (EE)		Model 2 (TI)		Model 3 (TI)	
	*β*	*t*	*β*	*t*	*β*	*t*
Gender	1.939	3.078**	0.079	0.456	0.331	1.749
RC	2.292	12.085***	0.377	6.066***	0.675	11.843***
EE			0.130	8.537***		
*R* ^2^	0.350		0.451		0.325	
*F*	85.499***		86.940***		76.648***	

RC = Role conflict; EE = Emotional exhaustion; TI = Turnover intention; **p < 0.01; ***p < 0.001.

Table 5 presents the results of the test of the moderated mediation effect on the relationship between role conflict and turnover intention. The interaction between role conflict and workplace relational conflict was found to be nonsignificant. This result suggests that workplace relational conflict does not moderate the relationship between role conflict and emotional exhaustion. Therefore, the fourth hypothesis proposed by this study is rejected. However, the results also found that workplace relational conflict moderates the effect of role conflict on turnover intention. Thus, the fifth hypothesis proposed by this study is supported. A summary of the hypothesis construct analysis can be found in Table 6 below.

**Table 5:** Testing the moderated mediation effect of role conflict on turnover intension.

Predictor	Model 1 (EE)		Model 2 (TI)	
	*β*	*t*	*β*	*t*
Gender	1.813	2.872**	0.095	0.588
RC	2.113	9.981***	0.220	3.587***
WRC	0.331	1.362	0.453	7.379***
EE			0.120	8.469***
RC*WRC	−0.124	1.093	0.095	2.149*
*R* ^2^	0.361		0.534	
*F*	44.559***		72.175***	

RC = Role conflict; EE = Emotional exhaustion; TI = Turnover intention; WRC = Workplace relational conflict; *p < 0.05; **p < 0.01; ***p < 0.001.

**Table 6:** Hypothesis construct analysis.

Hypothesis	Relation	*β*	Decision
H1	RC → TI	0.377***	Supported
H2	EE → TI	0.130***	Supported
H3	RC → EE → TI	11.843***	Supported
H4	RC → EE (moderated by WRC)	−0.124	Rejected
H5	RC → TI (moderated by WRC)	0.095*	Supported

RC = Role conflict; EE = Emotional exhaustion; TI = Turnover intention; WRC = Workplace relational conflict; *p < 0.05; ***p < 0.001.

## Discussion

The current study examined the impact of role conflict on turnover intention, the mediating effect of emotional exhaustion, and the moderating influence of workplace relational conflict among faculty members in Saudi Arabia. The results also found significant correlations between role conflict and turnover intention and between emotion exhaustion and turnover intention. In addition, the results indicated that emotional exhaustion significantly mediates the relationship between role conflict and turnover intention. Furthermore, the research presented here did not find any significant moderating effect of workplace relational conflict. However, there was a significant moderating effect on the mediated relationship between role conflict and turnover intention. Thus, four out of five hypotheses were supported, as shown in Table 6 above.

The results indicated a significant positive relationship between role conflict and turnover intentions (Hypothesis 1). Faculty members who struggle to balance their different roles were found to have more intentions to leave their institutions. This result is in line with the conclusions of previous literature, as several studies have found a correlation between role conflict and turnover intention among faculty members (Namin et al., 2021; Sadagheyani et al., 2021; Boamah et al., 2022) as well as a negative association with job commitment (Ababneh and Hackett, 2018). In addition, the findings identified the positive significant relationship between emotional exhaustion and turnover intention among faculty members (Hypothesis 2), which is consistent with the conclusions of previous studies (Sabagh et al., 2018, 2021; Namin et al., 2021; Boamah et al., 2022; Parmar et al., 2022). That is, faculty members who have a high level of emotional exhaustion make more attempts to quit their jobs or leave their institutions.

The results of this study are consistent with the conclusions of previous research regarding the mediating effect of emotional exhaustion on the relationship between role conflict and turnover intention (Hypothesis 3). Similar to the argument made by this study, Kulik et al. (2009) argued that employees who face many job demands ultimately deplete their resources, which causes them to experience burnout, one symptom of which is emotional exhaustion. A study conducted by Giao et al. (2020) investigated the impact of emotional intelligence on employee turnover and found a negative correlation between these factors. This result supports the findings of this study, as emotional exhaustion could be considered to be the opposite of emotional intelligence. Based on a moderated mediation model, Shin et al. (2020) found a positive correlation between role ambiguity and emotional exhaustion as well as a positive association between emotional exhaustion and turnover intention. Furthermore, Golu et al. (2022) identified emotional exhaustion as a mediator between work–family conflict, which could be considered to be a form of role conflict that can undermine the self. For employees, including faculty members, who are considering leaving their organizations, especially if they do not have another job, this behavior could be considered to be a form of self-undermining.

In this study, the hypothesized moderated mediation effect of workplace relational conflict on emotional exhaustion was rejected. This result is not consistent with the conclusions of previous research. The negative results of workplace relational conflict were found to be a stressor that decreases the resources available to employees, including faculty members. The researcher based his argument on two theoretical frameworks, namely, COR and job-demand theories. One explanation for this rejected result could be the fact that the faculty members who participated in this study already exhibited high levels of emotional exhaustion, and so they had already depleted all the resources that they could use to cope with role conflict. However, consistent with the fifth hypothesis of this study, a study conducted by Reio and Trudel (2013) reported negative correlation between workplace incivility and turnover intention. Workplace incivility is related to an escalation of relational conflict among employees in the workplace, which, as confirmed by this study, has a moderating effect on employees’ turnover intention. Similarly, Palanci et al. (2020) found a positive relationship between turnover intention and workplace relational conflict among employees.

## Conclusion

### Theoretical implications

The current study has several theoretical strengths and implications. First, this study is among the first to address the connections among role conflict, emotional exhaustion, turnover intention, and relational conflict among faculty members in a comprehensive way. Second, the fact that this study used validated scales to measure role conflict, emotional exhaustion, turnover intention, and relational conflict facilitated easier comparison to other reported data found by other international studies using the same scales. Third, this study was conducted in Spring 2021, i.e., during the period of the COVID-19 pandemic. Thus, this study arguably offers new perspectives on faculty members in the context of such extraordinary times.

### Practical implication

This study has several helpful practical implications. First, universities and institutions of higher education should make the necessary attempts to reduce role conflict and role overload. This goal could be achieved by developing more effective job design (Roczniewska and Bakker, 2021; Sahay et al., 2022), clearer job descriptions (Belias et al., 2021), and stronger policies and procedures (Malhotra et al., 2021). Second, faculty members’ emotional exhaustion should be reduced by providing training regarding conflict management (Esbati and Korunka, 2021), stress management (Kumar and Jin, 2022), and time management (Chen et al., 2021). Offering similar programs to faculty members could decrease stress resulting from their role conflict and lead to less emotional exhaustion, ultimately resulting in lower turnover intention. Third, early intervention and support for such faculty assistance programs (Wang et al., 2022) and counseling (Ghasemi et al., 2022) from universities and institutions of higher education are suggested to alleviate the negative impacts of role conflict and workplace relational conflict in this context.

### Limitations and directions for future research

Like any study, this study faces certain limitations. First, common method bias may be an issue for the current study, as the researcher used self-report measures for all variables. Future research can combine both faculty members’ self-assessments and assessments by the head of their academic departments to increase the validity of the results. Second, it is beyond the scope of this study to examine the actual turnover rate or turnover predictors and actual turnout behavior; rather, the researcher focused only on the turnover intention of faculty members. However, the researcher’s choice to focus solely on turnover intention was based on the suggestions made by previous turnover studies (Adams and Beehr, 1998; Blau and Lunz, 1998; Lee et al., 2000; Griffeth et al., 2016), which have argued that turnover intention is more effective than other turnover predictors. Future research can investigate other turnover-related predictors or behaviors to provide a broader understanding of turnover. Third, this study used relational conflict as the only component of interpersonal conflict. It would be interesting to determine whether adding other components of interpersonal conflict, such as cognitive conflict, would change the outcomes of this study. Thus, future research is encouraged to investigate this issue further. Fourth, this study was conducted in Saudi Arabia and examined only one profession, namely, faculty members. Future studies can examine the conceptual model developed in this study in the context of different populations and professions to provide more generalizable insights into this topic. Finally, since this research is a quantitative cross-sectional study, a characteristic which might be viewed as a limitation, future studies can conduct longitudinal and/or qualitative studies to gain a better understanding of and broader perspectives on this topic.

## Data availability statement

The raw data supporting the conclusions of this article will be made available by the authors, without undue reservation.

## Ethics statement

Ethical review and approval was not required for the study on human participants in accordance with the local legislation and institutional requirements. The patients/participants provided their written informed consent to participate in this study.

## Author contributions

The author confirms being the sole contributor of this work and has approved it for publication.

## Conflict of interest

The author declares that the research was conducted in the absence of any commercial or financial relationships that could be construed as a potential conflict of interest.

## Publisher’s note

All claims expressed in this article are solely those of the authors and do not necessarily represent those of their affiliated organizations, or those of the publisher, the editors and the reviewers. Any product that may be evaluated in this article, or claim that may be made by its manufacturer, is not guaranteed or endorsed by the publisher.
